# Improved comprehension of influenza‐related headaches: Perspectives and suggestions for incidence and prevalence of headache in influenza—Response

**DOI:** 10.1111/ene.16478

**Published:** 2024-09-30

**Authors:** David García‐Azorín, Laura Santana‐López, Ana Ordax‐Díez, José Eugenio Lozano‐Alonso, Diego Macias Saint‐Gerons, Yésica González‐Osorio, Silvia Rojo‐Rello, José M. Eiros, Javier Sánchez‐Martínez, Álvaro Sierra‐Mencía, Andrea Recio‐García, Ángel Luis Guerrero‐Peral, Ivan Sanz‐Muñoz

**Affiliations:** ^1^ Department of Medicine, Faculty of Medicine Universidad de Valladolid Valladolid Spain; ^2^ Headache Unit, Department of Neurology Hospital Clínico Universitario de Valladolid Valladolid Spain; ^3^ Dirección General de Salud Pública e Investigación, Desarrollo e Innovación, Gerencia Regional de Salud Junta de Castilla y Leon Valladolid Spain; ^4^ Department of Medicine University of Valencia, INCLIVA Health Research Institute and CIBERSAM Valencia Spain; ^5^ Department of Microbiology Hospital Clínico Universitario de Valladolid Valladolid Spain; ^6^ National Influenza Centre Valladolid Spain; ^7^ Fundación Instituto de Estudios de Ciencias de la Salud de Castilla y Leon ICSCYL Soria Spain

One of the strengths of this study was the systematic and consistent evaluation of a series of symptoms when patients seek medical attention at primary care [1]. This was done through a clinical in‐person interview by a trained healthcare provider. Since headache was one of the evaluated symptoms, its incidence and prevalence could be assessed.

Khan et al. provided a series of suggestions that we acknowledge and would like to comment on [2]. In our understanding, the use of medication could have influenced the prevalence of headaches in the following ways: (a) symptomatic treatment could have decreased the duration of the headache, and by the time patients were evaluated in primary care the headache might not be present; (b) headaches could have started after the medical evaluation [1]. However, according to other studies, headaches seem to be an early symptom that typically resolves within 4 days [3]. If so, the true prevalence of headaches could be underestimated. Regarding the use of antiviral therapies, these require a medical prescription, so it is not possible that these could have modified the prevalence of headaches. It would be interesting for future studies to evaluate whether these therapies may modify the clinical phenotype and/or duration of headaches.

Regarding the role of epidemiological contacts and travel history, as an epidemic disorder it is common that the number of cases peaks during certain periods of the year when the transmission of the virus is higher within the population [1]. According to the literature, the main factors that seem to influence the virulence are the specific circulating strains and variants and the proportion of vaccinated individuals [4]. With more than 8000 studied patients and 12 influenza seasons, the picture seems comprehensive enough to evaluate possible annual differences, which did not seem to be that remarkable regarding headache epidemiology [1].

Concerning the probability of seeking medical attention, we believe that Khan et al. [2] refer to another publication from the same study [5]. In that study, as Khan et al. [2] point out, we did not prospectively follow up with patients, and we were only able to compare whether patients with headaches were referred to hospitals more or less frequently. It was observed that patients with headaches had 54% lower odds of being referred to the hospital, and it seemed clear that this was not observed by chance [5]. The reasons are not known with certainty. However, we hypothesize that the reason may be a more efficient immune response. Patients with headaches also had a higher frequency of other symptoms commonly associated with the immune response and the release of cytokines and interleukins. This has been observed with other acute viral infections, and we aim to validate it in future studies [5, 6].

We agree that prior influenza infections could play a relevant role, and not only these but also vaccination status, the circulating strain, and prior contact with other viral infections. It seems that immune memory against influenza does not last for life, and the circulating viruses vary from 1 year to another, with regional and national differences [4]. This was observed in our study, where patients infected by B subtypes had a higher frequency of headaches. In contrast, vaccination status did not yield any differences [1].

Regarding ‘psychological states’, stress and anxiety can trigger headaches in patients with pre‐existing headaches, either primary headache disorders or secondary headaches. Primary headache disorders are highly prevalent, but their prevalence was lower than that observed in our study [7]. Psychiatric manifestations, delirium or hallucinations were out of the scope of the present study, which specifically focused on headaches, but in our opinion do not seem to explain the commonly experienced headaches in most patients.

## CONFLICT OF INTEREST STATEMENT

D.G.‐A. has received honoraria for lectures/presentations from AbbVie/Allergan, Eli Lilly, Teva, Lundbeck, and Novartis. D.G.‐A. has participated in clinical trials as the principal investigator for Pfizer, BioHaven, and Lundbeck. D.G.‐A. is junior editor of The Journal of Headache and Pain. D.G.‐A. has received honoraria from the World Health Organization as a subject matter expert. Á.L.G.‐P. has received honoraria for lectures/presentations from AbbVie/Allergan, Eli Lilly, Teva, Lundbeck, and Novartis. Á.L.G.‐P. has participated in clinical trials as the principal investigator for Eli Lilly, Teva, AbbVie, Novartis, Amgen, and Lundbeck. None of the other authors has any conflict of interest to disclose.

## Data Availability

The data that support the findings of this study are available on request from the corresponding author. The data are not publicly available due to privacy or ethical restrictions.

## References

[1] García‐Azorín D , Santana‐López L , Ordax‐Díez A , et al. Incidence and prevalence of headache in influenza: a 2010–2021 surveillance‐based study. Eur J Neurol. 2024;31(8):e16349. doi:10.1111/ene.16349 38770742 PMC11236060

[2] Khan S , Hussain JM , Khalid AB , Ramsha S . Improved comprehension of influenza‐related headaches: perspectives and suggestions for inicidence and prevalence of headache in influenza. Eur J Neurol. 2024;12:1009.10.1111/ene.1647739264226

[3] García‐Azorín D , Santana‐López L , Lozano‐Alonso JE , et al. InfluenCEF study: clinical phenotype and duration of headache attributed to influenza infection. Cephalalgia. 2023;43(11):3331024231212900. doi:10.1177/03331024231212900 37950674

[4] Brody H . Influenza. Nature. 2019;573(7774):S49. doi:10.1038/d41586-019-02750-x 31534258

[5] García‐Azorín D , Santana‐López L , Lozano‐Alonso JE , et al. Factors associated to the presence of headache in patients with influenza infection and its consequences: a 2010–2020 surveillance‐based study. J Headache Pain. 2024;25(1):18. doi:10.1186/s10194-024-01728-z 38331709 PMC10854039

[6] Trigo J , García‐Azorín D , Planchuelo‐Gómez Á , et al. Factors associated with the presence of headache in hospitalized COVID‐19 patients and impact on prognosis: a retrospective cohort study. J Headache Pain. 2020;21(1):94. doi:10.1186/s10194-020-01165-8 32727345 PMC7388434

[7] Stovner LJ , Hagen K , Linde M , Steiner TJ . The global prevalence of headache: an update, with analysis of the influences of methodological factors on prevalence estimates. J Headache Pain. 2022;23(12):34. doi:10.1186/s10194-022-01402-2 35410119 PMC9004186

